# A case of bleeding shock induced by injury of the intercostal artery following percutaneous nephrolithotripsy

**DOI:** 10.1002/iju5.12657

**Published:** 2023-10-24

**Authors:** Hiroaki Kakinoki, Yukako Yamaguchi, Minika Yukimoto, Yuka Kakinoki, Kazuma Udo, Shohei Tobu, Go Takeshita, Yoshiaki Egashira, Ken Yamaguchi, Mitsuru Noguchi

**Affiliations:** ^1^ Department of Urology, Faculty of Medicine Saga University Saga Japan; ^2^ Department of Radiology, Faculty of Medicine Saga University Saga Japan

**Keywords:** bleeding shock, embolization, intercostal artery, percutaneous nephrolithotripsy

## Abstract

**Introduction:**

The risk of postoperative bleeding complications should be concerned to perform percutaneous nephrolithotripsy. Most of the vascular injuries occurred at the peripheral renal artery in the previous reports. We experienced a case of bleeding shock induced by the injury of the intercostal artery in the abdominal wall following percutaneous nephrolithotripsy.

**Case presentation:**

A 56‐year‐old woman had been in the bleeding shock status on the 2nd day after percutaneous nephrolithotoripsy. Emergently, contrast‐enhanced computed tomography was performed and extravasation of contrast agents was seen in the abdominal wall. Injuries of the intercostal artery were identified in the angiography and controlled by transcatheter arterial embolization.

**Conclusion:**

The intercostal arteries could be injured in the anterolateral zone of the abdominal wall over the end of the ribs. Contrast‐enhanced computed tomography was useful to detect the bleeding point. Transcatheter arterial embolization was an effective and safe method to control bleedings from them.

**Abbreviations & Acronyms**
CECT: contrasted‐enhanced computed tomography
PNL: percutaneous nephrolithotripsy
TAE: transcatheter arterial embolization

**Keynote message**

The injury of the intercostal artery rarely occurred to perform PNL, but it could cause severe bleedings. It can be harmed in the anterolateral zone of the abdominal wall over the end of ribs. CECT and TAE were useful for its management.

## Introduction

PNL is recommended as the prior option for large renal stones above 20 mm and staghorn stones. The rate of postoperative bleeding complications is higher than ureteroscopy and extracorporeal shockwave lithotripsy.^1^ The acute hemorrhages associated with PNL often occurred at the renal parenchyma and TAE was effective for hemostasis.^2^

The intercostal artery could be often injured by blunt traumas and the medical intervention for the chest,^3^, ^4^ but the injuries following urologic surgeries were rare. This is a case report about the bleeding shock induced by the injury of the intercostal artery in the abdominal wall following PNL.

## Case presentation

**Fig. 1:**
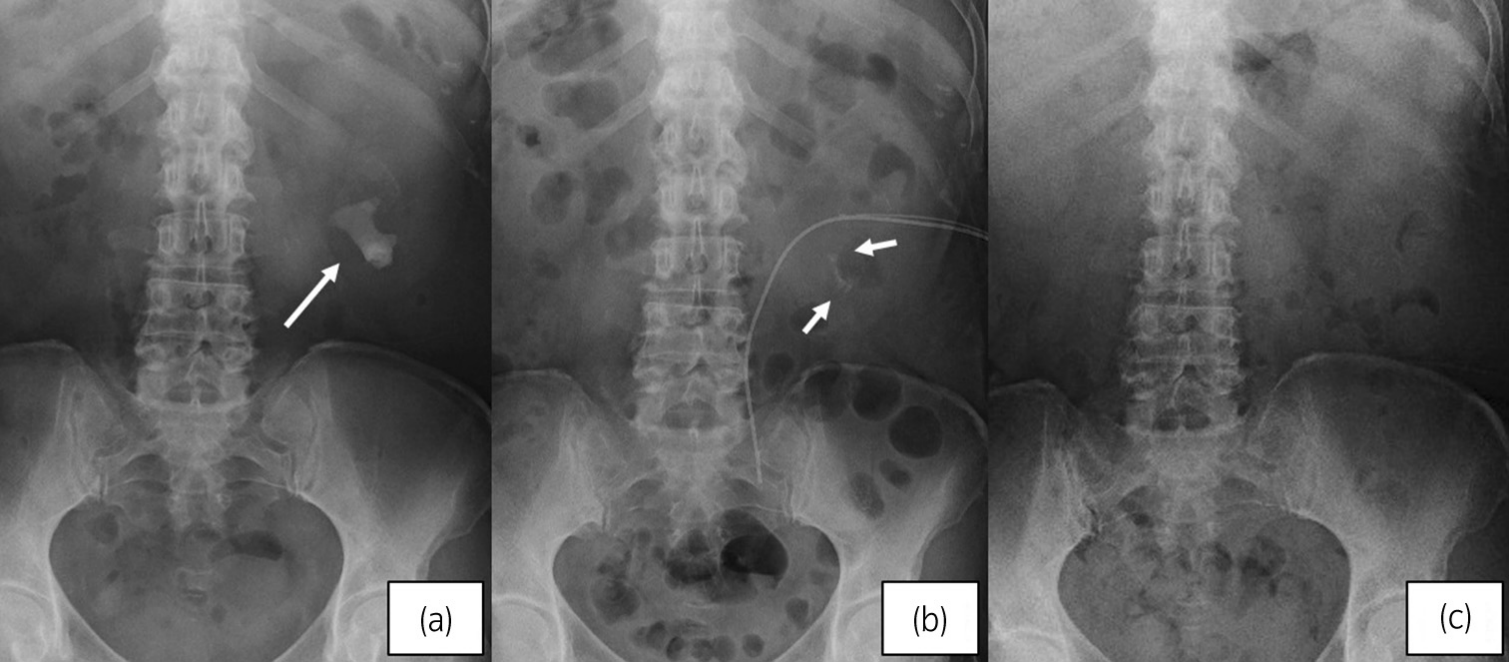
Abdominal X‐ray. (a) The arrow indicates the staghorn stone which was 43 mm as long diameter in the renal pelvis and lower calix before PNL. (b) The arrows indicate the dust and multiple residual fragments smaller than 3 mm diameter in the lower calix on the next day after the first PNL. (c) The stone fragments were completely removed after the 2nd PNL.

A 56‐year‐old woman with staghorn calculi with 43 mm as long diameter in the abdominal X‐ray (Fig. 1a) was referred to our hospital. She had the comorbidities of hypertension, diabetes mellitus, obesity (Body Mass Index was 33), and without coagulopathy.

**Fig. 2:**
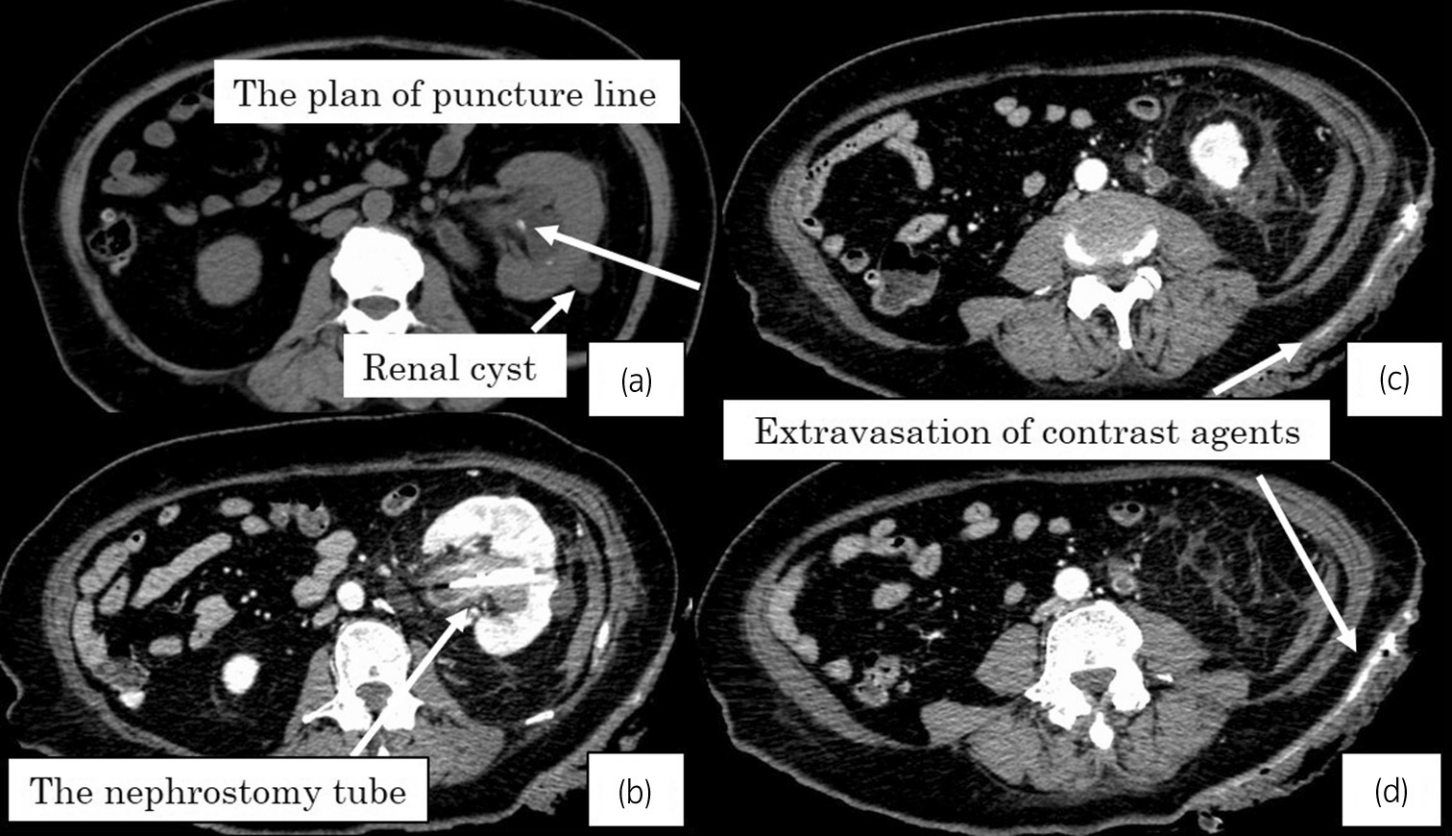
(a) Pre‐operative non‐contrast computed tomography (CT); The long arrow indicates the planned puncture line into the dorsal‐middle calix in the anterolateral zone compared with usual, because we should avoid the renal cyst (the short arrow). (b–d) Contrast‐enhanced CT after the bleeding. (b) The arrow indicates the nephrostomy tube. There was no active bleeding around the left kidney. (c,d) The arrows indicate the extravasation of contrast agents in the abdominal wall.

The surgical procedure was as following. The patient was set in the Galdakao‐modified‐Valdivia‐position^5^ under general anesthesia. We planned to puncture into the dorsal‐middle calix along the anterior line to avoid the renal cyst (Fig. 2a). 0.035‐inch guidewire was successfully inserted into the urinary collecting system at the first puncture of the 18 gauge needle guided by ultrasound and fluoroscopy. The 24Fr Amplatz sheath was inserted through the tract which was enlarged by metallic telescopic serial dilator. The stone was crushed by Holmium laser (Lumenis VersaPulse PowerSuite 30W: 0.5–0.6 J/ 5–15 Hz) and removed by forceps visualized by the rigid and flexible nephroscope. There was no bleeding which prevent the endoscopic view during the surgery. The 20Fr nephrostomy tube with the 5Fr ureteral catheter to prevent slip‐out was indwelled at the end. The operation time was 97 min. The dust and multiple fragments smaller than 3 mm diameter were seen in abdominal X‐ray on the next day of PNL (Fig. 1b), and hemoglobin drop was not seen (11.9 g/dl).

Suddenly, massive bleeding was seen around the nephrostomy tube on the 2nd day after PNL. CECT was examined after 37 min from onset of the bleeding. The extravasation of contrast agents was seen in the abdominal wall (Fig. 2b–d). It could not be controlled by finger pressure and suturing around the nephrostomy. Then, the patient had got into the hemorrhagic shock status (the blood pressure was 76/44 mmHg and hemoglobin dropped to 9.1 g/dl).

**Fig. 3:**
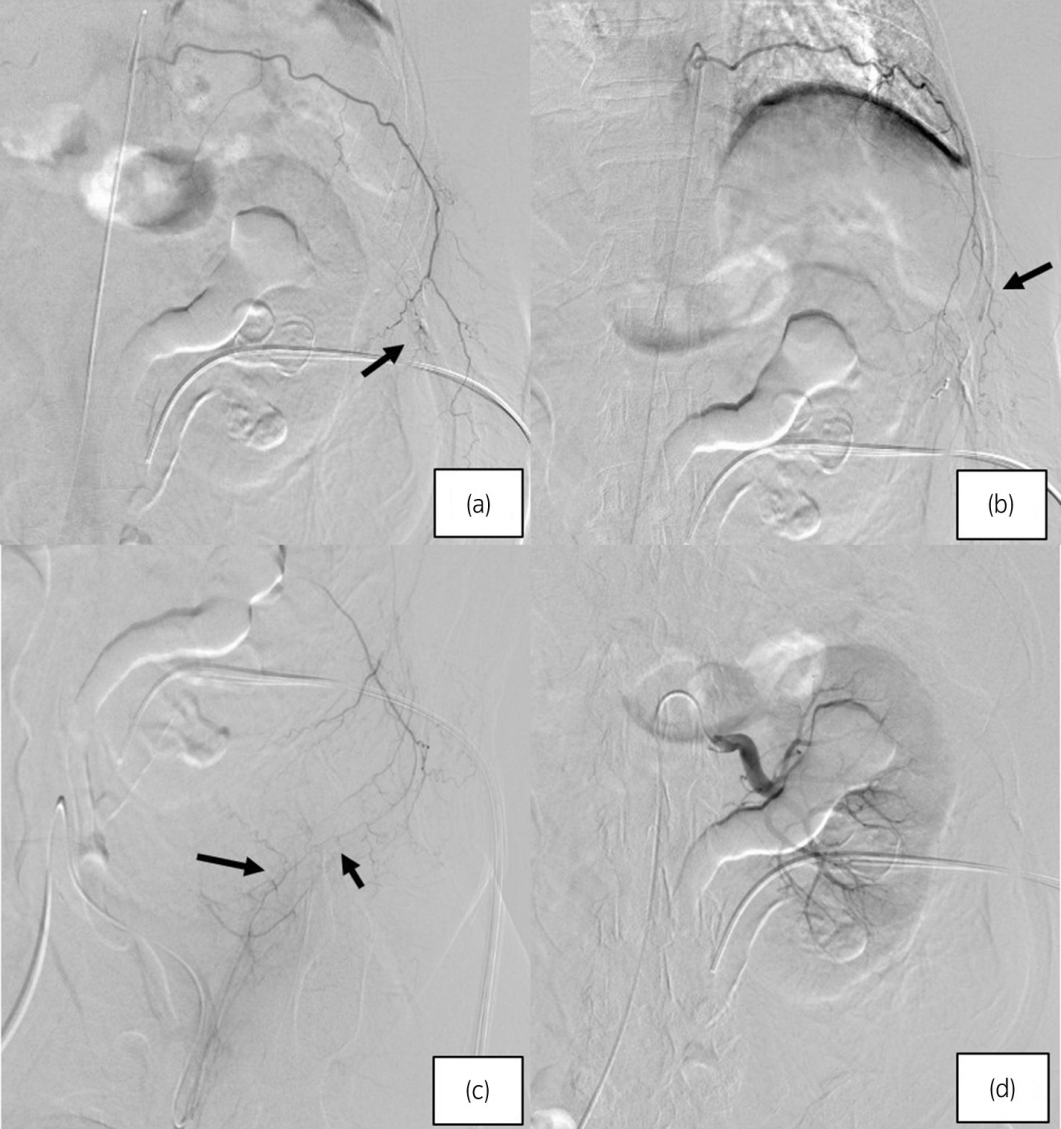
TAE was performed emergently. (a,b) The arrows indicate active bleeding at the distal branches of the 10th and 9th intercostal arteries which embolized by gelatin sponges. (c) The arrows indicate a communicating branch between the 10th intercostal artery and the left circumflex iliac artery which was also embolized. (d) There was no active bleeding in the renal angiography.

Emergently, TAE was performed by the interventional radiologist after 123 min from onset of the bleeding. The 4Fr sheath was inserted into the right femoral artery by the Seldinger technique under local anesthesia. The injuries of the distal branches of the 9th and 10th intercostal arteries were identified and embolized by gelatin sponges cut into 1 mm size through the micro‐catheter involving the communicating branch of the circumflex iliac artery (Fig. 3). The blood pressure had been recovered to 97/65 mmHg as soon as closing TAE. A lot of infusion (normal saline, 3000 ml, and albuminous preparations, 5% 500 ml) were intravenously dripped to stabilize hemodynamics. On the next day of TAE, the hemoglobin dropped down to 7.2 g/dl and the transfusion of red blood cells (560 ml) was needed.

On the 7th day after PNL, the patient got a high fever. Pseudomonas aeruginosa was detected in her blood and urine sample. Ceftriaxion and ceftazidime were intravenously administered for 7 days. The 2nd PNL by flexible nephroscope and basket retrieval device was performed on the 19th day (operation time was 51 min), and she could discharge without residual fragments on the 30th day from the first PNL (Fig. 1c).

## Discussion

The bleeding complications associated with PNL occurred as the rate of transfusion was 7% and embolization was 0.4%.^1^ Most cases of bleeding were reported as pseudoaneurysm or arteriovenous fistula due to the injuries of the peripheral renal artery. TAE is an effective and safe method to control them.^2^

The intercostal arteries were often injured by blunt traumas and medical interventions in the previous reports. Thoracentesis, chest‐drain positioning or removal, Hepatic radiofrequency, cardiac surgery, thoracotomy, and percutaneous biliary procedures were reported as the majority of iatrogenic factors.^3^, ^4^ We could find only two cases of hemothorax secondary to the injury of the intercostal artery following PNL by the supracostal approach. R. Gupta *et al*. concluded that the supracostal approach could provide a high clearance rate with acceptable complications (chest complication was 5% and hemothorax was 2%) in their report.^6^, ^7^

**Fig. 4:**
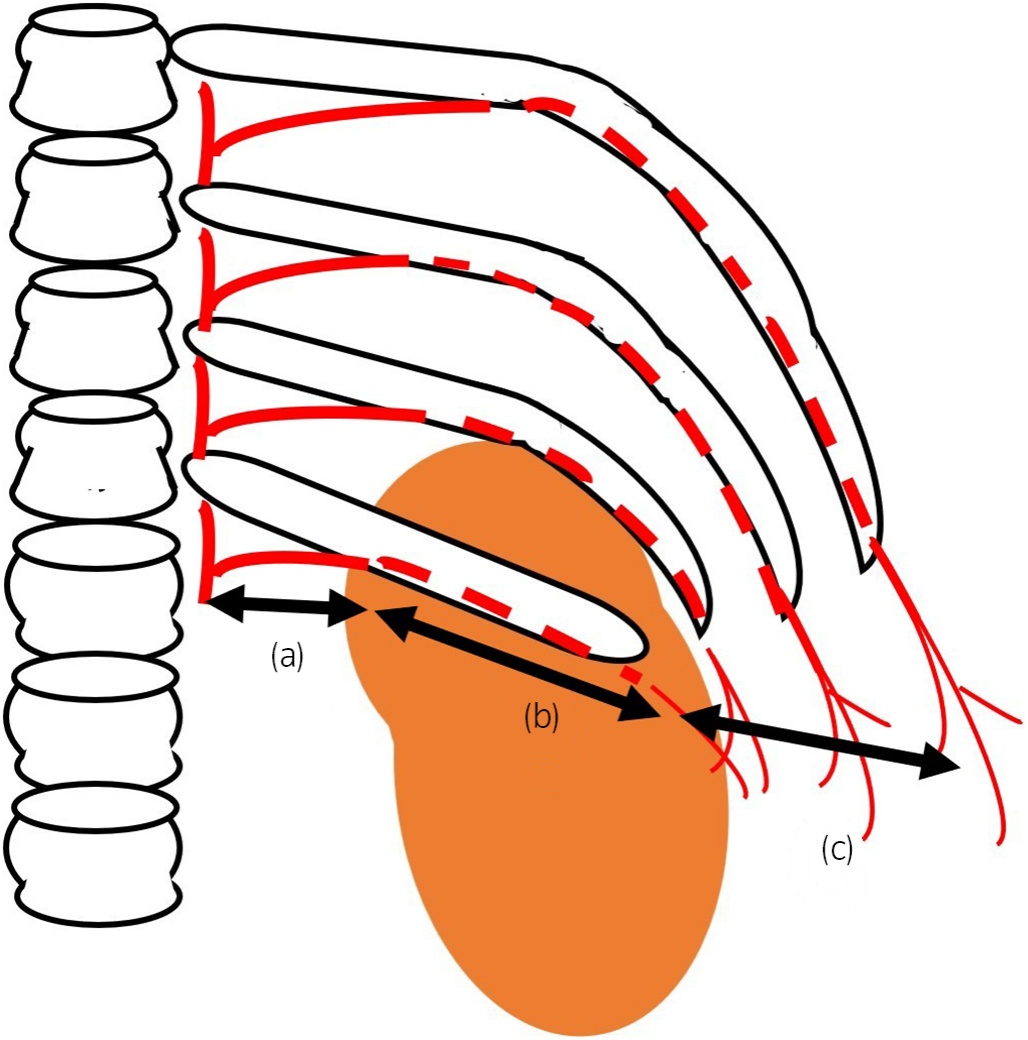
Anatomy of the intercostal arteries. (a) The intercostal arteries are apart from superior ribs at the central zone of the first 6 cm near to the spine. (b) The neurovascular bundles with the intercostal arteries are shielded by the intercostal grooves at the lateral zone over 6 cm from the spine. (c) There are multiple branches of the distal intercostal arteries which are feeding into surrounding skins and muscles at the anterolateral zone over the end of ribs without the bone shield effect.

We should know about the anatomy of the intercostal arteries. Helm *et al*. reported that the intercostal arteries were apart from superior ribs at the zone of the first 6 cm near to the spine (Fig. 4a) and shielded by ribs at the far lateral zone over 6 cm^8^ (Fig. 4b). In this case, we needed to puncture into the calix at the anterolateral zone of the abdominal wall, because the abundant perinephric fat with obesity elevated the axis and we had to avoid the renal cyst which was close to the puncture line (Fig. 2a). As a result, there might be higher risk of harming the distal branch of the intercostal artery where is over the end of the ribs and without the bone shield effect (Fig. 4c). B area where the intercostal artery is shielded by the rib is probably safer to puncture and access than A and C area. Identification of the intercostal arteries by color‐Doppler‐ultrasound could be a safe method to avoid injuries of them.^9^ Furthermore, mini PNL with the smaller tract (12–20Fr scale) had been reported about its advantage in reducing bleeding complications.^10^

CECT was useful in identifying the bleeding point. TAE was performed safely without ischemic complications at the abdominal wall and the spinal cord and effective in controlling the acute massive bleeding from the intercostal artery in the abdominal wall.

## Author contributions

Hiroaki Kakinoki: Conceptualization; writing – original draft. Yukako Yamaguchi: Writing – review and editing. Minika Yukimoto: Writing – review and editing. Yuka Kakinoki: Writing – review and editing. Kazuma Udo: Writing – review and editing. Shohei Tobu: Writing – review and editing. Go Takeshita: Writing – review and editing. Yoshiaki Egashira: Writing – review and editing. Ken Yamaguchi: Writing – review and editing. Mitsuru Noguchi: Supervision.

## Conflict of interest

The authors declare that have no conflict of interest.

## Approval of the research protocol by an Institutional Reviewer Board

Not applicable.

## Informed consent

Informed consent was obtained.

## Registry and the Registration No. of the study/trial

Not applicable.
